# Performance Evaluation of Triangulation Based Range Sensors

**DOI:** 10.3390/s100807192

**Published:** 2010-07-29

**Authors:** Gabriele Guidi, Michele Russo, Grazia Magrassi, Monica Bordegoni

**Affiliations:** 1 Department of Industrial Design, Art, Communication and Fashion (INDACO)—Politecnico di Milano, Via Durando 38/A, 20158, Milan, Italy; E-Mail: michele.russo@polimi.it (M.R.); 2 Department of Mechanical Engineering - Politecnico di Milano, Via La Masa 34, 20156, Milan, Italy; E-Mails: grazia.magrassi@polimi.it (G.M.); monica.bordegoni@polimi.it (M.B.)

**Keywords:** metrological characterization, 3D measurement, laser scanner, pattern projection, resolution, uncertainty, accuracy

## Abstract

The performance of 2D digital imaging systems depends on several factors related with both optical and electronic processing. These concepts have originated standards, which have been conceived for photographic equipment and bi-dimensional scanning systems, and which have been aimed at estimating different parameters such as resolution, noise or dynamic range. Conversely, no standard test protocols currently exist for evaluating the corresponding performances of 3D imaging systems such as laser scanners or pattern projection range cameras. This paper is focused on investigating experimental processes for evaluating some critical parameters of 3D equipment, by extending the concepts defined by the ISO standards to the 3D domain. The experimental part of this work concerns the characterization of different range sensors through the extraction of their resolution, accuracy and uncertainty from sets of 3D data acquisitions of specifically designed test objects whose geometrical characteristics are known in advance. The major objective of this contribution is to suggest an easy characterization process for generating a reliable comparison between the performances of different range sensors and to check if a specific piece of equipment is compliant with the expected characteristics.

## 1. Introduction

**Figure 1.**
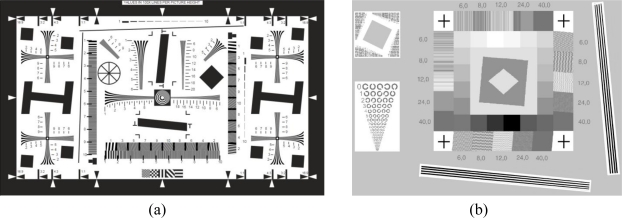
Standard targets for 2D imaging equipment characterization: **(a)** ISO12233 target for directly measuring horizontal, vertical and oblique resolution of a digital camera and frequency domain analysis; **(b)** ISO 16067 target for measuring spatial resolution of a 2D scanner for reflective material.

It is well known that the performances of an imaging system are associated with several parameters, which depend on the kind of sensor that is considered. In 2D imaging, for example, various standards have been defined for photographic equipment [1] and bi-dimensional scanning systems [2], based on specific targets and on the related procedures for estimating different system parameters (e.g., the spatial resolution in terms of lines per millimeter, the amount of noise, image compression and gamma correction functions *etc.*). The test charts presented in Figure 1 represent an example of such targets.

In the last two decades, due to the rapid growth in performances of digital technologies, 3D imaging has seen significant advances with a corresponding decrease in costs, which has made three-dimensional acquisition and modeling more and more widespread in several application areas, ranging from industrial manufacturing to cultural heritage documentation [3]. But differently from 2D imaging, no standard test protocols currently exist for evaluating the performance of 3D imaging systems.

Therefore, the testing procedures adopted by the 3D imaging industry are not univocally defined and the official technical features for end-users are not often easily comparable among them. For this reason, in order to take full advantage of 3D imaging systems some metrological laboratories have been set up in the last few years, pointing out not only the advantages, but also the limitations of these instruments. Besides the creation of specific laboratories, research studies are being conducted in various research centers aiming at defining a characterization processes for assessing 3D camera features.

This paper lies in this stream, and is focused on investigating an experimental process for creating a 3D equipment datasheet with quantitative and comparable data. The creation of standards and the definition of neutral and coherent test methodologies are critical for increasing user awareness in the application of 3D acquisition technologies. Although these standards should address the whole measuring process from acquisition to data processing [5], in this research the attention has been focused on the 3D acquisition device, employing a “black box” approach, where a global evaluation of some functional parameters, depending on the superposition of optical and electronic effects, is given.

The paper presents a short overview of previous works in the field of 3D equipment characterization in section 2. Section 3 is devoted to a general description of the proposed methodology, Section 4 analyzes the equipment tested and their nominal operating characteristics, Section 5 describes material and geometry of the custom target objects used in this experimentation, with a particular attention to their certification, Section 6 shows the experimental results organized according to the parameter under analysis. Conclusions and perspectives about this characterization approach are reported in the last section.

## 2. Previous Work

In the 3D imaging domain, the definition of best practices that led to standards started less than 15 years ago [4]. Measurement accuracy and uncertainty related to the various 3D imaging systems have been reported for specific applications in a metrological laboratory with controlled conditions [5], or for practical usage in less controlled conditions, with emphasis on triangulation based range devices [6]. For Time of Flight (TOF) based devices, a specific set of tests has been reported by Bohler *et al.* regarding both accuracy and resolution in standard conditions [8], and by Guidi *et al.* for their use at low scanner-to-target distance [9].

A few applications of the concept of Modulation Transfer Function (MTF) have been developed for determining the resolution level of a 3D imaging system and applied in the biomedical imaging area for X-Ray devices [10] and mammotomography equipments [11]. In the field of laser based range cameras, the MTF method was first applied by Goesle *et al.* [12] by making the comparison between the MTF of an acquired sharp corner and the corresponding theoretical shape.

Metrological definitions are not yet shared and accepted in the 3D field, and this has led to the creation of specific working groups aiming at creating a metrological standard for 3D optical devices [13], whose work is still in progress.

## 3. Proposed Methodology

The methodology presented in this paper is based on the production of 3D images related to specifically developed 3D test objects, whose size and shape are known in advance, with a level of accuracy far better than the parameters we intend to evaluate. Such accuracy has been obtained by modeling each object with a CAD tool, manufacturing it with a suitable technology as, for example, Computer Numerically Controlled (CNC) machine tools and, independently of the specific manufacturing process, measuring each physical prototype with a Coordinate Measurement Machine (CMM) capable of giving an accuracy of below 4 micrometers in the xyz directions, while the parameters we intend to evaluate range from 20 to 300 micrometers (*i.e.*, five times or more the CMM accuracy).

The accuracy of each piece of equipment has been estimated by comparing the actual dimensions of each certified object with the measures attainable through their range maps, acquired with the non-contact range devices under test. Since some parameters were too dependent on the sampling pattern generated in any acquired range image, we decided to make wide use of primitive fitting in order to get each parameter as the result of an averaging process rather than a single measured value.

A clear example is the measurement of a cone height, which can be surely wrong unless the tip of the cone corresponds exactly with one of the 3D points acquired by the range device. Conversely, if a set of points representing a cone is fitted with a conical primitive, the cone height is determined with a high level of accuracy, independently of the sampling grid positioning, by measuring the height of the corresponding primitive. The deviation of such value from the cone height determined by CMM measurements will be the accuracy range device estimation for that particular parameter. This approach has been applied for each geometrical shape used.

Uncertainty estimation has been referred to both planar and curved surfaces. It has been obtained by calculating the standard deviation of each acquired range map with respect to the corresponding ideal geometries. We have used this approach since 3D devices may give different response when the optical axis is inclined with different angles to the explored area. For example, let us consider a set of points representing a cylinder acquired through a device oriented at right angles to the cylinder axis; the deviation of the actual 3D points from the corresponding primitive will be different in the areas where the device’s optical axis is perpendicular to the cylinder surface, while it will be progressively different as the angle changes. For this reason, both standard deviations given by a plane and by a curved surface are separately reported in the results.

Finally, the resolution has been estimated differently along the optical axis of each range device (z) and along the other two directions (xy). These two resolution parameters will be indicated hereafter as z resolution and horizontal resolution respectively. One key point of the proposed approach for resolution estimation consists in representing the range images associated with each 3D device as grey level images on which the analyses previously developed for the 2D imaging area can be applied. For example, if a black to white transition has to be replicated, the corresponding 3D target will be characterized by an abrupt jump of its 3D surface along the z direction, and the range device will be oriented in order to have its optical axis parallel to that direction, centered on the 3D feature. The range map will be thus transformed in a grey level image by assigning white and black to the minimal and maximal sensor-to-target distance respectively, with points of distances inside this range linearly grey coded. The resulting 2D image can be processed by means of ISO processes as those defined in ISO 12233 and ISO 16067. With such approaches the resolution can be estimated in two ways:

- *Directly*: through a set of 3D features that are progressively closer to each other. The resolution is defined by detecting at which spatial frequency such features are not distinguishable anymore to a common observer;
- *Indirectly*: through the analysis of the system response, in the frequency domain, to a wideband stimulus such as a step, which is represented in a grey level image as an abrupt black to white transition.

In our experiments, the horizontal resolution has been estimated in the latter way, while z resolution has been explored through a specific 3D object characterized by several steps of progressively smaller values, evaluating up to which level these were detectable on the range image.

Specific range maps have been acquired for each test object by using homogeneous nominal settings, in order to characterize the aforementioned parameters in comparable operating conditions.

The software packages used for extracting the parameter of interest from the range maps consist of specifically developed MATLAB procedures, public domain MATLAB scripts (sfrmat2), and commercial packages for both 3D data processing (Innovmetric Polyworks) and for checking resolution estimation (QuickMTF).

## 4. Tested Instruments

Differently from TOF range sensors, where each point of a range map is generated by measuring the time needed by a light pulse for going from the emitting sensor back to a photodetector, in triangulation range sensors the geometrical information is generated thanks to a scan head made by a light source generating one or multiple sheets of light, and projecting them at known angles on the target. A video camera, positioned at a fixed baseline from the source, acquire the corresponding light profiles and generates 3D data of the points belonging to each profile by estimating their parallax (*i.e.*, their distance from the central axis of the image sensor). Once the 3D data of a profile are estimated, by mechanically moving the scan head an entire 3D image is generated. As an alternative, if a sufficiently high number of light planes is directed to the target, as with pattern projection cameras, a 3D image can be directly generated without moving elements (*i.e.*, full frame cameras) [3].

**Figure 2.**
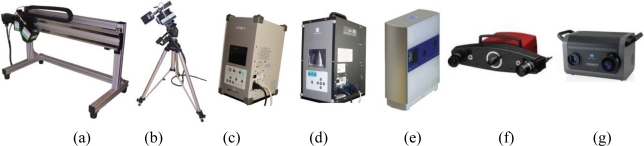
Set of 3D laser scanner technology used in our experiment, from left to right: **(a)** SG100 (ShapeGrabber Inc.), **(b)** SG1000 (ShapeGrabber Inc.), **(c)** Vivid910 (Minolta), **(d)** V9i (Minolta), **(e)** NextEngine (NextEngine Inc.), **(f)** Athos (GOM), **(g)** Range7 (Minolta).

The instruments used for our experiment are shown in Figure 2 and consist of seven triangulation range devices available in our laboratories at Politecnico di Milano: the Reverse Modeling Lab (INDACO Dept.), and the HapRE Lab (Mechanical Engineering Dept.).

The instruments are listed below with their main characteristics:

- ShapeGrabber® SG100: this laser line triangulation scanner is equipped with a high precision linear rail system that permits a 60 cm horizontal translation of the sensor side by side. The minimum working distance (Standoff) is 90 mm while the maximum is 290 mm. The focal length is fixed and each profile is made by 1,280 3D points.
- ShapeGrabber® SG1000: this is provided with a mechanical rotating head that permits a 330° rotation along its axis. The standoff is 250 mm, and the maximum working distance is 1,150 mm. The focal length is fixed and each profile is made by 1,280 3D points.
- Minolta® Vivid 910: includes three lens with different focal length: Tele (f = 25 mm), Middle (f = 14 mm) and Wide (f = 8 mm). It has a working distance ranging from 600 mm to 2,500 mm and is provided with a bi-dimensional CCD (640 × 480 pixel), that defines a fixed sampling grid on the imaged surface.
- Minolta® Vivid VI-9i: this is a model derived from the Vivid 910, provided with updated opto-electronic equipment but with the same 640 × 480 sensor size.
- Nextengine®: this is based on the Multistripe Laser Triangulation (MLT) technology. The 3D camera is equipped with a twin array of 4 solid state lasers and two 3 Mpixel CMOS RGB array sensors. The system makes the acquisitions in two different modes that correspond to two different baselines: wide mode, which requires the object to be 45 cm far from the front of the scanner, and the macro mode, where the object is positioned 16 cm far away. We have used the instrument in the Wide mode configuration and Fine modality, to assure a nominal resolution level compliant with the other devices.
- GOM® Atos II: is a pattern projection range sensor based on the triangulation principle. It is equipped with one pattern projector and two cameras with fixed focal lens (f = 17 mm) observing the object to be measured from different points of view in order to reduce the effects of occlusions.
- Minolta® Range7: \is a laser stripe device provided with two lenses: Tele (f = 25 mm) and Wide (f = 8 mm). The reflected light is received by a CMOS sensor that evaluate 3D information by acquiring 1.31 megapixels (1,280 × 1,024) images of the laser light profiles. The working distance ranges from 450 to 800 mm.

The instruments set-up of each device was chosen in order to have approximately the same sample spacing along the horizontal plane (0.3 mm). Sometimes this was not possible, as for example for the SG100, made for high resolution measurements at close range, where the minimal sampling step along the scan-line is 0.1 mm. In this case the choice was to set up the only parameter changeable (*i.e.*, the profile spacing). The actual set-ups are reported in Table 1.

## 5. Test Objects

**Figure 3.**
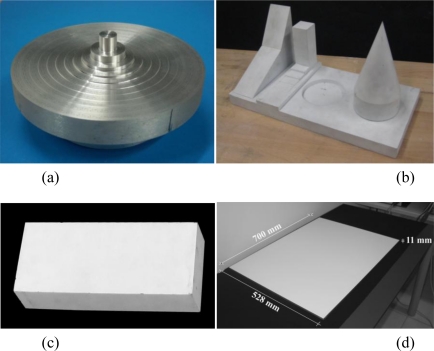
Test objects used in the experiments: **(a)** set of steps for testing z resolution and accuracy; **(b)** set of solids for testing accuracy and uncertainty; **(c)** parallelepiped block for testing xy resolution; **(d)** reference plane for testing uncertainty and relative accuracy.

In order to reduce measuring time and expenses, we have defined some targets with different geometries using standard materials, which we were able to produce using the facilities available in our laboratories (see Figure 3). For avoiding light reflections, producing signal saturations in range devices processing that are converted in geometrical artifacts superimposed on the “true” range data, the test objects used in this paper have been matt white painted in order to make their surfaces as diffusive as possible. Only the surface of one of them was not painted in order to avoid any geometrical alteration of the smaller 3D features. The actual objects are described in detail below.

### 5.1. Set of Step

The first object is a “set of steps” (Figure 3a). It is a set of coaxial cylinders, carved form a single block of iron, with diameters varying linearly from 100 mm to 10 mm, at steps of 10 mm. Its height changes not linearly from 15 micrometers up to 7.68 millimeters, with a doubling of the step size for each transition (*i.e.*, 15, 30, 60, 120, 240, 480, 960, 1920, 3840, 7680 micrometers). The object has been manufactured by using a CNC milling machine (Biglia CNC B301). As the other test objects specifically manufactured for this research, it has been measured before the 3D scanning tests with a mechanical CMM Zeiss Prismo 5 Vast HTG MPS (according to ISO 10360-2: u1 = 1.5 + L/350 μm; u3 = 2.0 + L/300 μm, with L expressed in mm), which gave us the reference parameters and dimensions for our studies. The temperature in the lab was kept at 20 °C with a maximum temperature deviation of +/−1 °C in order to minimize the influence of possible thermal variations in size of our metallic test objects. From this inspection the object shown slight deviations values, with a max height of 7,672 μm (8 micrometers deviation of the real object from the CAD model). The actual height of each step was used for testing resolution and accuracy along the optical axis direction (z). Therefore the surface of the object was not treated with any paint layer in order to avoid any geometrical alteration at the smaller steps. The nominal and actual size of each step is reported in Table 2.

### 5.2. Set of Solids

The second target is a set of solids mechanically connected to a thick plate, and painted matte white to avoid reflections. As shown in Figure 3b (from left to right), it includes two planar surfaces, one inclined with respect to the other, a set of steps, a cylindrical volume carved in the basis, and a cone coaxially superimposed on a cylinder with a basis having the same diameter. The overall dimension of the plate is 400 mm × 200 mm × 200 mm.

We have investigated the accuracy and uncertainty of the 3D data generated from these characteristic features, such as the cone slope, the cylinder diameter, and the angle between the two intersecting planes. The other parts of the object have not been used because they presented geometrical features similar to other test objects but less suitable for our tests.

### 5.3. Parallelepiped Block

This target is a parallelepiped block, made of rectified iron painted matte white to avoid reflections. It has dimension 100 mm × 60 mm × 270 mm and its main feature is to become a 60 mm vertical step, once laying over an horizontal planar reference with its larger face. Such step shape has been used as large bandwidth stimulus to the systems under test in order to investigate their xy resolution performances with a frequency domain analysis described in section 6.3.

### 5.4. Reference Plane

In order to guarantee small deviations from planarity and low cost, we have decided to use a thick (11 mm) piece of glass. The particular manufacturing process of this material allows one to obtain a planar target with a peak deviation from the theoretical plane in the order of few micrometers. Geometrically, this suitable for testing our devices, characterized by measurement uncertainties and accuracies in the range of 20–400 micrometers. Optically, this material is not as suitable as it is geometrically, due to its transparency. This property makes it not compliant with an active range sensor, which works only of diffusive reflecting surfaces. We solved that by applying matte white paint on one side of the target. It was dried with an oven treatment, with the same process used for painting cars, to avoid as much as possible the generation of geometrical irregularities on the paint layer. This solution permitted to obtain, at low cost, a good planar and optically cooperative surface. The area of this planar reference (700 mm × 528 mm) was large enough for covering the acquisition field of all the instruments tested.

## 6. Tests and Experimental Results

In this section we present the experimental approaches used to obtain accuracy, uncertainty and resolution from some range images taken in pre-defined conditions with all the devices under test, applied on the test objects mentioned above, starting from the “a priory” knowledge of their actual geometrical data.

### 6.1. Uncertainty Analysis

This parameter is defined as the dispersion of the z coordinate of each point (target-to-sensor distance) around its average value. In other words, it represents the random component of the measurement error. This particular parameter can be estimated by calculating the distance of each measured point from its theoretical value, which is here given by the point on the corresponding fitted primitive. The standard deviation extracted from the statistical distribution of this sequence of distances gives an evaluation of the instrument uncertainty. The targets used for this test are both the planar reference and the set of solids.

#### 6.1.1. Reference Plane

**Figure 4.**
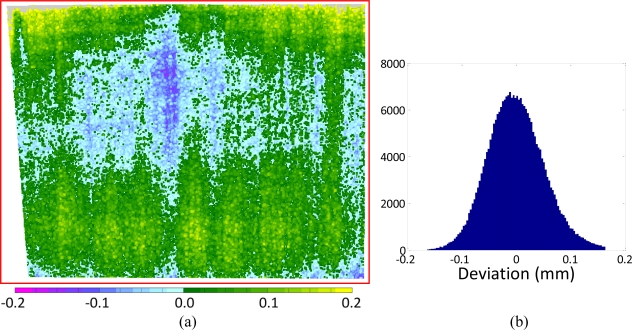
Comparison between the range data originated by a laser scan of the planar reference made with the Minolta V9i scanner (25 mm lens) and the corresponding best fitting plane: **(a)** error deviation color coded in the range −0.2 ÷ 0.2 mm; **(b)** histogram of the random errors showing a nearly Gaussian shape.

Range maps generated by acquiring the planar reference through each range device, have been processed numerically in order to find the equation of the corresponding best fitting planes. The residual deviations between the actual measured points and the fitting plane, which was assumed to be the mathematical representation of the true physical reference, were first imaged and then statistically processed calculating the error histogram and the corresponding standard deviations [7], as shown for example in Figure 4.

An image like that in Figure 4a allows to make evident that in the same range map we always find a deviation from the theoretical behavior due to measurement errors, that can be seen as the superposition of a random component, expressed by the grainy texture of the image, and a systematic component, associated for example with the blue spot at the center and the green areas in the upper and lower parts of the image.

In the histogram of Figure 4b a nearly Gaussian behavior is evident, due to the fact that most of such random deviation is generated by the analog electronic processing chain before image digitization, known as CCD read noise. Here the thermal noise, whose distribution is well known to be Gaussian, has a dominant role [14].

In addition, a pseudo-random component might be added on the laser based devices for the speckle effect. It is produced by the reflection of the coherent laser light on diffusive surfaces that, for their intrinsic roughness, give to any reflected photon a different phase contribution. This generates a destructive or constructing interference on the backscattered light when it reaches the recording sensor, depending on the actual position over the sensor area. Such effect involves a Gaussianly-distributed random parallax alteration between points at the same sensor-to-target distance that once added to the electronic noise effect, maintains the same statistical distribution. Seven different range maps corresponding to each analyzed 3D scanner have been analyzed, obtaining the uncertainty evaluation reported in Table 3.

**Figure 5.**
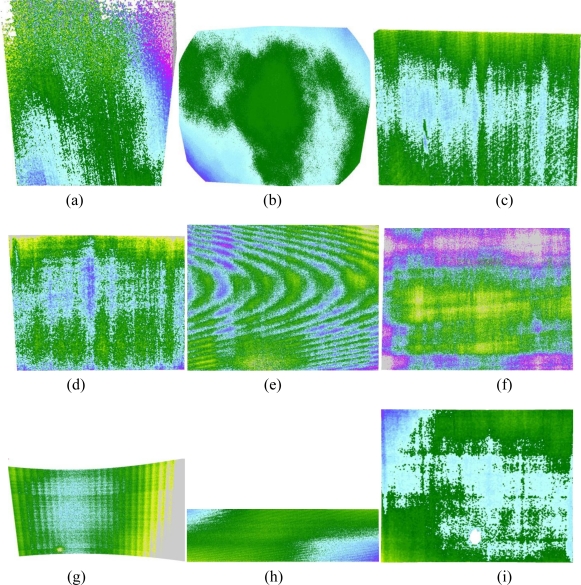
Deviations from the fitting plane of the range devices under test, color coded as in Figure 4a: **(a)** Nextengine; **(b)** GOM Atos II; **(c)** Minolta VI-9i 14 mm; **(d)** Minolta VI-9i 25 mm; **(e)** Minolta VI-910 14 mm; **(f)** Minolta VI-910 25 mm; **(g)** SG1000; **(h)** SG100; **(i)** Minolta Range7.

The deviation from the Gaussian behavior of the error histograms in Figure 4b is mainly due to the clear superposition of the systematic error to the purely random one. This add a fluctuation in the average of the zero mean Gaussian random phenomenon associated with noise and speckle. Such fluctuation involves a growth in the standard deviation, as shown by the difference between the standard deviation σ_1_, evaluated by fitting a plane on the whole framed area and calculating the statistics of residual 3D points-to-fitting plane deviations, and σ_2_, evaluated on a 50 mm × 50 mm subarea in the center of the range image. In addition the same table reports the peak positive and negative deviation of each data set from the fitting plane, once outliers were manually eliminated (e.g., wrong 3D measurements due to reflection or clearly recognizable artifacts). This might also be considered indicator of the systematic error influencing σ_1_, which after all can be considered as a cumulative index of measurement uncertainty and accuracy. On the other hand systematic alterations in most acquisition appear to be characterized by a very low spatial frequency. As shown in Figure 5, with the exception of VI-910 with 14 mm lens (Figure 5e), whose factory calibration (not changeable by the end-user) seems to be rather poor, all the maps make evident a systematic gradual change from green to blue that most of the times involves the whole field of view, in the range of tens of centimeters. On the other hand σ_2_, being evaluated on a 50 mm × 50 mm central spot of each range map can be considered as an uncertainty index, purely due to random effects.

#### 6.1.2. Set of Solids

The object described in Figure 3b was acquired with two range maps for each 3D device. One range image was aimed at acquiring the two inclined planes keeping the device as perpendicular as possible to both planes. This set-up was chosen in order to have approximately the same σ alteration due to optical axis-to-plane angles on the upper and lower plane, that has been proved to be angle dependent [9].

A second range map was generated for acquiring the cone superimposed on the cylinder, keeping the camera axis approximately perpendicular to the cone axis. For both acquisitions the standard deviation (1σ) between the actual data and the fitting primitives (cone, parallelepiped, planes, *etc.*) have been evaluated and reported in Table 4.

### 6.2. Accuracy Analysis

In any measurement device the concept of accuracy refers to the systematic component of the measurement error with respect to the real data.

We define here as *linear accuracy* of a specific range device the absolute deviation between a linear dimension known in advance and accepted as the true value (e.g., the radius of a cylinder, the height of a cone or the distance between a reference plane and any measurable three-dimensional feature in the space), and the same linear dimension obtained through a range image generated by that device.

Similarly, we define as *angular accuracy* of a specific range device the absolute deviation between an angle known in advance and accepted as the true value (e.g., the angle between two intersecting planes, the angle between the generator and the axis of a cone, *etc.*), and the same angle obtained through a range image generated by that device.

Both aforementioned parameters are represented by single values even if originated by a cloud of 3D points, because of the extraction of these data is based on a fitting primitive, that operates an averaging of several raw 3D points for producing the actual single value (e.g., a cylinder fitted on a set of 3D points is a way for extracting one clean diameter value from several 3D noisy points).

Finally we define here *relative linear accuracy* as the deviation of a set of 3D points from the shape they should describe (rather than from their actual position in space) divided by the diagonal of the range map. Typical example is the 3D scan of a plane, where all the points should be coplanar, but for a number of reasons they are displaced from such theoretical behavior, as shown in Figure 4a. Since such deviation has sign, we indicate as relative linear accuracy the following value:

$$A_{\mathrm{LR}}=\frac{\Delta z_{\max}+\mathrm{abs}\left(\Delta z_{\min}\right)}{d}$$

where Δz_max_ and Δz_min_ are the maximum and minimum deviation of the actual range data from the fitted primitive expressed in micrometers and d is the diagonal of the framed area expressed in millimeters. Differently from the two previous parameters, given by an averaging process, this one is evaluated directly on the raw data, taking into account the worst samples, after the elimination of outliers. This parameter represents in practical terms the average accuracy error along z (in micrometers), for each millimeter explored by the range map along the xy plane.

All accuracy parameters have been extracted from range maps, using as test objects both the set of solids and the set of steps, as described in the next two sections.

#### 6.2.1. Reference Plane

From the peak positive and negative errors reported in Table 3, the relative linear accuracy has been easily calculated estimating the absolute error by the difference between the columns Δz_max_ and Δz_min._ and dividing the result for the diagonal size of each analyzed image. The values reported in Table 5 exhibit a remarkable good performance for the GOM Atos II, with only 0.31 of relative linear accuracy, less than one third the second better result (Minolta Range 7). Then Minolta 9i in its two configurations and the two ShapeGrabber represent another group of coherent accuracy performances ranging between 1.21 and 2.50, followed by the Minolta 910 with both lenses, and finally the NextEngine.

It can be noticed that linear accuracy is always better with short focal lengths for both scanners usable with different focal lengths.

#### 6.2.2. Set of Solids

For checking the capability to catch the actual value of an angle between surfaces inclined each other, the range map of the set of solids has been acquired and processed.

**Figure 6.**
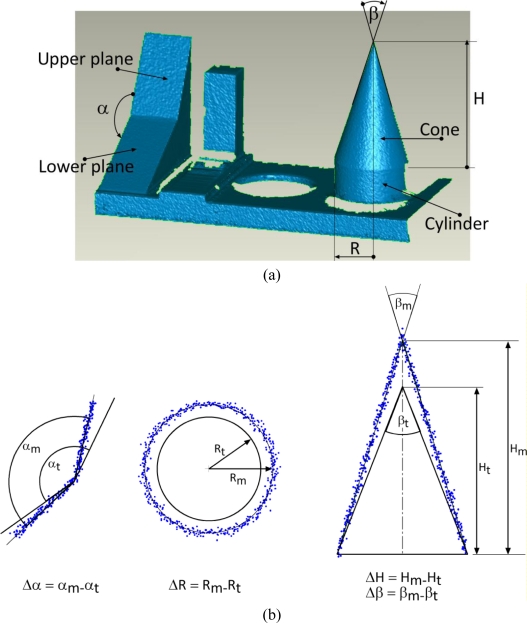
Components under analysis for the “set of solids” test object: **(a)** naming given to the different geometrical elements; **(b)** definition of measurement deviations Δα, ΔR, ΔH and Δβ, related respectively to the angle between planes, the radius of the cylinder, the height of the cone and its generator inclination. In this pictorial representation the measurement uncertainty has been deliberately exaggerated in order to make clear the meaning of such parameters.

The angular accuracy has been measured in two different ways. First we have evaluated the angle α between the two intersecting planes shown in the left part of Figure 6a by selecting, for each plane, groups of points belonging to it, and evaluating the corresponding fitting planes. The angle between such planes is the angle measured through the range map; it is indicated in Figure 6b as α_m_. The value of the same parameter assumed as “true”, and indicated as α_t_, is the one generated through the CMM measurements. The deviation Δα has been evaluated as difference between the measured and the true values, as shown in Figure 6b.

Secondarily, the angle defined by the tip of the cone, corresponding to twice the angle between the generator and the axis of the cone has been evaluated. Similarly to the previous angle, the set of data acquired on the cone surface has been used for fitting a conical primitive. The resulting cone slope, indicated in Figure 6b as β_m_, was considered as the angle originated by the measurement. Its deviation from the value assumed as true, given by the CMM, has been defined as Δβ.

Finally, the linear accuracy in this situation has been estimated by comparing the cylinder radius and the cone height extracted from the conical primitives associated with the acquired 3D data, and the corresponding CMM values.

As we can see from the values reported in Table 6, the angular deviations are in general rather low, with some larger values for the angle between planes. Here the most significant angular deviations, above 0.2 degrees as absolute value, are for the two Minolta V9i and V910 with the 25 mm and 14 mm respectively. Conversely, angular estimations lower or equal than 0.11 degree are obtained with all the other devices, with a very accurate angular performance for SG100 and Range7.

The linear accuracy is extremely good for GOM Athos II in both cone height and cylinder radius estimation. The first parameter has in general larger deviations, anyway below half millimeter for NextEngine, VI-910 with the 14mm lens and for the Minolta Range 7. Regarding the cylinder radius the best results were obtained with Minolta Range 7. An absolute error below 50 micrometers was obtained with all the other devices, with the exception of SG1000, that evidenced an error more than three times larger of the second worst device.

#### 6.2.3. Set of Steps

**Figure 7.**
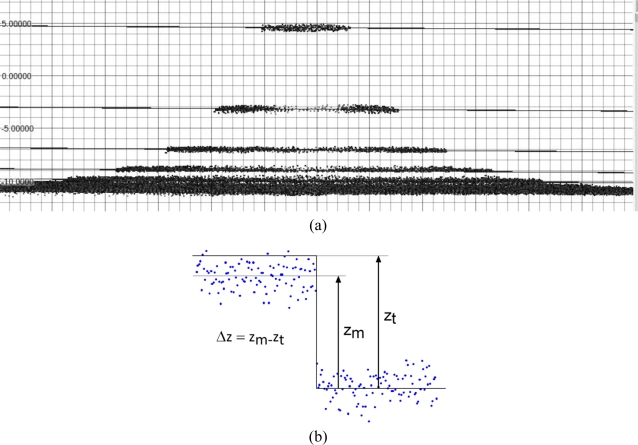
“Set of steps” experiment: **(a)** lateral view of a range map generated by 3D acquisition of the test object and planes associated with the different steps; **(b)** measured step size value *vs.* true value.

From the range map of this kind of test object we have been able to evaluate the linear accuracy along the z direction. At first a reference planar primitive has been created starting from the wider ring (higher sensor-to-object range) and used as zero reference. Then, for each step, a plane parallel to the zero reference has been created and located at a distance defined by the CMM measurements reported in section 5.1. The set of 3D points belonging to the specific set were then selected and the average distance between those points and the related plane was evaluated (Figure 7) and reported in Table 7.

Since this selection was made by hand, this process made evident the need of distinguishability of points belonging to a specific step, which was not the same for the different range devices (see 6.3).

### 6.3. Resolution Analysis

Two different resolution aspects have been examined: limitation in z-resolution due to the opto-geometric configuration (distance-to-baseline ratio), and horizontal resolution capability mainly due to optical performances.

In the first case, the “set of steps” object has allowed us to test the z resolution. Range maps of the sequence of steps have been acquired with different sensors. The z resolution capacity of every instrument has been detected by means of the user’s capability of identifying and selecting the set of points belonging to each step of the object. This activity has been necessary for calculating the root mean square distance between the selected points and the primitive defined by CMM measures (see accuracy analysis). When the points that should lie on the same plane are not distinguishable from those belonging to the adjacent step, that point is classified as an undistinguishable step; the last distinguishable step is defined as the z resolution limit.

The xy resolution has been estimated by acquiring a sharp edge of the metallic parallelepiped. The edge of the range map has been transformed into a B/W image by coding the distance (z) as a gray level. The ISO slanted edge analysis has been applied on the resulting image [1]. The resolution capability of the optical system based on an image with slanted edge can be found by evaluating the SFR (Spatial Frequency Response) behavior, also called MTF (Modulation Transfer Function).

#### 6.3.1. Set of Steps

The z resolution test involved a check on the range maps originated by acquiring the set of steps test object with all the different devices under test. The same set of data were used to verify the linear accuracy along optical axis.

The selection of circular sets of 3D points corresponding to the different steps was made according to their distance from the object axis. However, the influence of uncertainty and accuracy on the actual optical resolution of each device imposed a well defined criteria for deciding if a set of 3D points is distinguishable from the adjacent ones.

The relationship between the average given by a certain set and the value assumed as true seen in the previous section, would suggest the possibility to detect instrumentally a distinction between sets even when characterized by very small z differences.

For example deviations below 10 micrometers in the detection of all steps have been found, an amount lower than the smaller step to be detected (16 μm). Although this instrumental possibility, since this method is the 3D counterpart of the direct ISO resolution detection, we decided to leave to an operator the final decision about the distinguishability of each subset of data, depending on the color-coded deviation map of the acquired data compared to a plane located in correspondence of the first ring of data (zero reference).

**Figure 8.**
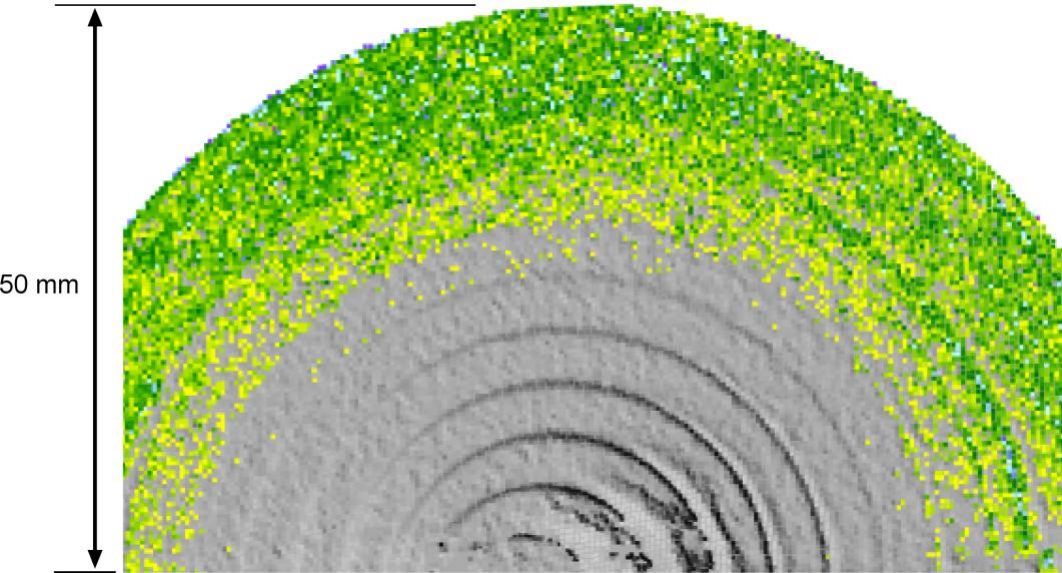
Color coded deviation of the range map originated by the acquisition of the set-of-steps with a Minolta Vi with 14 mm lens respect to the reference plane obtained by best-fitting the first plane (zero reference). It appears clear that with this scanner the first two steps are not recognizable.

Figure 8 shows such deviation map for the Minolta V9i equipped with the 14 mm lens. As clear in the image the superposition of local unaccuracies documented in section 6.1, together with the typical Gaussian uncertainty of such data and the actual optical resolution, led to a deviation map that make clearly distinguishable by a naked eye only the third step of this set, and not the smaller ones on the upper part of the image.

**Figure 9.**
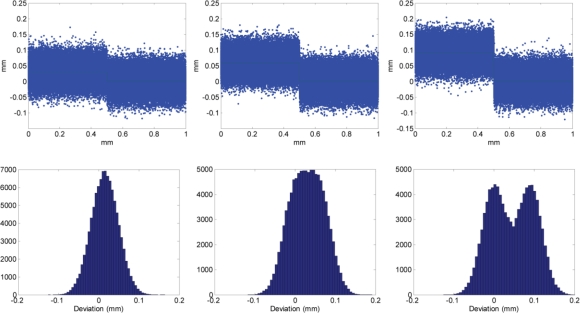
Simulated 3D acquisition of a step 30, 60 and 90 μm high (from left to right), with a measurement uncertainty of 30 μm. Raw data are in the upper row, corresponding frequency histograms in the lower one. Groups of data are statistically undistinguishable in the first two cases.

The same test was performed with different color maps and range of analysis, basically with the same result. This is probably due to the randomness of 3D data, expressed by the standard deviations reported in Table 3, that, even if superimposed on clearer data (like the ones simulated in Figure 9), tend to hide the variations of the average value underneath.

In order to exemplify this concept, a simulation of a set of random data Gaussianly-distributed around a predefined z level, have been considered, with a standard deviation α of 30 micrometers. In this way 10,000 points have been generated, half around z = 0, and half around z = step, with three different step height, given by σ, 2σ, and 3σ respectively, and statistically processed as a whole set of data. As highlighted by Figure 9 for the first two steps the corresponding histogram has a nearly Gaussian behavior, with no evidence of grouping, as evidenced instead by the third case (step = 3σ). Although the analysis made by an operator tends to be focused on the average color rather than on the samples dispersion around the average, the latter fact generates a level of noise that tends to hide such tiny z-jump. The step estimation becomes even more difficult when the unavoidable effect of relative accuracy shown in Figure 5 is superimposed on the actual data. The “operator based” approach is a way for taking into account the cumulative effect of all phenomena described above on the z resolution.

Using the range data processing described above for checking z-accuracy it is possible to see directly which is the minimal z-deviation that appears detectable to the average user. With reference to the symbols reported in Table 6, the z resolution for each scanner may be estimated as the first distinguishable z-jump. Therefore it is 16 μm for ShapeGrabber SG100 and Minolta Range7, 60 μm for GOM Atos II, Minolta VI-9i (Middle), Minolta VI-9i (Tele), Minolta VI-910 (Middle), Minolta VI-910 (Tele) and ShapeGrabber SG1000, and 239 μm for the NextEngine.

#### 6.3.2. Parallelepiped block (3D Slanted Edge Analysis)

In addition to the “set of steps” target, used here for a direct estimation of resolution along the z axis, the xy resolution of a 3D system can be tested indirectly by analyzing its output in the frequency domain.

The concept of frequency is easily understandable in acoustics, where a deep voice is associated to a low frequency, while a high note corresponds to a high frequency. The frequency, in that case, represents the number of cycles of an acoustic wave in the time lag of one second, and therefore the measurement unit is the inverse of time (1 Hz = 1 /sec).

In the optical case we consider spatial frequency, with reference to the cyclic repetition of image details rather than sound waves. If we consider for example an image pattern made of a sequence of vertical black and white strips, we can evaluate the maximum number of transitions in a fixed space actually acquirable by an optical digital system, as the measurement of its capability to digitize the complex iconographic content of an image.

This capability is influenced both by the optical resolution of the lens, and by the bandwidth of the electronic system that will operate the amplification and the A/D conversion of the sensor signal. As in an electro-acoustical chain (*i.e.*, CD, amplifier and loudspeakers), the sound quality is determined by the frequency range that the sequence of devices is capable to reproduce without attenuation, similarly, in the optical field, we can evaluate the range of spatial frequencies that the system is able to acquire, by analyzing its Spatial Frequency Response (SFR). This function corresponds to the module of the Optical Transfer Function (OTF), also known as Modulation Transfer Function (MTF). In order to highlight that such spectrum is referred to a spatial frequency rather than a temporal frequency, the measurement unit, instead of Hertz, is given in terms of number of transitions from black to white in the space of a millimeter, or line pairs per millimeter (lp/mm). For digital sensors, instead of referring the number of cycles to an absolute length (1 mm), such frequency can be expressed in relative terms as cycles per image pixel (cy/px), neglecting in this way the absolute size of the acquisition sensor.

The practical way for evaluating the MTF of an optical system consists in estimating the capability to reproduce an abrupt luminance variation, by analyzing an acquired image containing a black-to-white transition, in the frequency domain.

**Figure 10.**
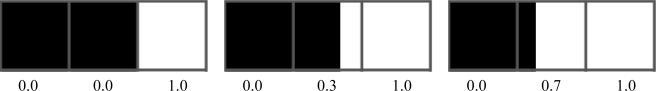
Pixel values (0 = black, 1 = white) originated on an horizontal line by different sampling grid positioning (gray line) respect to an abrupt black to white transition.

Given that the image sensor is digital, the black-to-white transition may involve a different number of pixels depending on the relative positioning between the pattern and the sampling grid. This is why the edge is not chosen for example exactly vertical. In this case the sampling would in fact remain the same in all the image lines. A slanted edge ensure a different relative positioning of the black-to-white transition respect to the sampling pattern, as shown in Figure 10 for an ideal optical system, generating no blurring on the image.

Since the spectral analysis for estimating the MTF, is made on the transition, in order to average the effect of the relative grid-pattern positioning on several pattern rows, a pattern made with a straight slanted edge is used, attenuating in this way possible artifacts induced by any specific sampling.

**Figure 11.**
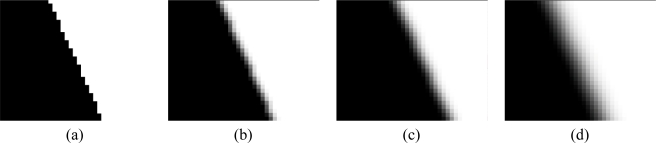
Example of slanted-edge with a blurring level progressively stronger: **(a)** No blurring; **(b)** 0.5 pixel mask; **(c)** 1.0 pixel mask; **(d)** 2.0 pixel mask. The edge has been zoomed in this figure for the sake of intelligibility. No re-sampling has been done on the raw images.

In order to make evident the relationship between the amount of blurring and the related MTF, in the Figure 11 slanted edge synthetically generated with a graphic package has been analyzed, together with different smoothed versions obtained with a “blurring filter” employing a filtering mask of progressively growing size (0.5, 1.0, 2.0 pixels).

**Figure 12.**
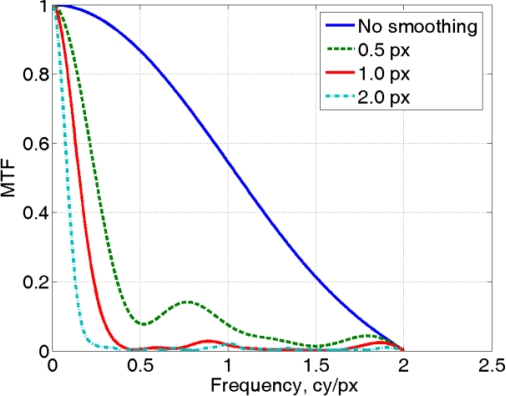
Plot of MTF related to the four simulated cases described in Figure 11.

As shown in Figure 12, the spectrum is much wider in the first case since the number of spectral components present in image 11a is high, while for increasing blurring values the spectrum width decreases, because the range of spatial frequencies present in images 11b, 11c and 11d is progressively reduced.

In addition to this qualitative explanation of the relationship between MTF and image sharpness, a quantitative parameter can be conventionally defined as the number of cycles per pixel (or line pairs per millimeter), corresponding to 50% of the spectral peak (MTF50).

The evaluation here described can be done with public domain software, as the MATLAB script sfrmat2, developed by Peter Burns (Kodak) [15,16], or with other commercial packages, as for example Imatest (http://www.imatest.com). The corresponding resolution values are reported in the following Table 8, showing a smaller spectrum width as the slanted edge blurring increases.

This concept can be transferred to 3D sensors, by converting a range map in a gray level image, where each camera-to-target distance is coded with light gray levels for points close to the camera and darker gray levels, as the detected points become farer.

The slanted edge can be generated with a parallelepiped, as the one shown in Figure 3c, lying over a reference plane, and the 3D image can be acquired keeping the range device axis at right angles to the reference plane. The range map attainable with such acquisition can be transformed in B/W image, and, from the MTF analysis of the latter, the xy resolution can be estimated.

The oblique straight line generated through the parallelepiped object is useful for ensuring a multiplicity of sampling grid positioning over the black-white transition, shown for the 2D image in Figure 10, as defined by the ISO standards for image capture devices evaluation [1,2].

It is important to notice that the image generated from the range map has a 1:1 correspondence between 3D points and image pixels, with no re-sampling. In order to have the best possible correspondence between device performances and image indexes, any post-processing on range maps, such as smoothing, cleaning, *etc.*, was also avoided. For this reasons it was not possible to check two of the range devices under test, due to the automatic post-processing activated at file export on those devices.

**Figure 13.**
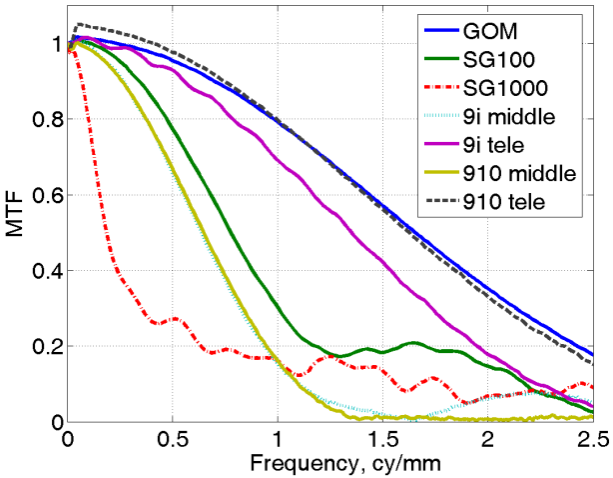
Plot of MTF related to the seven combinations of 3D scanner/lens analyzed in the paper.

In Figure 13, the set of MTF associated with the different scanners analyzed in the paper, are reported. The larger spectra represent the better resolution (GOM), while the narrower give the coarser (Minolta VI910-Middle and ShapeGrabber SG1000). The corresponding MTF50 values are reported in Table 9.

## 7. Conclusions

In this paper we have presented a low cost method for characterizing 3D cameras. Specifically, the use of ISO derived approaches have allowed us to easily obtain results by using methods that have been already accepted in other domains. In the experimentation, seven different pieces of equipment with nine configurations, nominally similar in performance, have given very different results, thus evidencing the need of standard parameters for characterizing a 3D camera.

Accuracy, uncertainty and resolution parameters have been evaluated during an experimental phase that involved specific certified test objects. Angular accuracy appears to be good for each range sensor (below 7% errors in the worst case), while linear accuracy seems more influenced by the range sensor cost. In particular the parameter Relative Linear Accuracy, easily evaluable in a lab with a reference plane, gives a quality classification of the devices under test that has a correspondence with the quality “perceived” by expert users and with the equipment cost.

The measurement uncertainty evaluated on planes ranges from 5 to 60 micrometers and is roughly coherent with what declared by each manufacturer. The same parameter, when evaluated on a cylinder reveals an amazing difference between different range devices. This is due to cameras capability in maintaining a good capture quality with the camera axis very angled respect to the surface to be captured; for example, a very good uncertainty for Minolta Range 7 and GOM on the plane (5 and 8 μm respectively), on the cylinder becomes 18 μm for the Range 7 (2×) and 159 μm for the GOM (32×).

At last the obtained parameters show a qualitative and quantitative evaluation of horizontal and z resolution. The cheapest system that we have tested (NextEngine) cannot give a readable result up to the fifth step of the “set of steps” test object, while two of the most expensive (Minolta Range 7 and GOM Atos II), have deviations smaller than 20 micrometers for the whole range of 10 steps. The evaluation of xy resolution seems to be clearly discriminated thanks to the transposition of the MTF based approach to 3D imaging.

These kinds of tests represent a preliminary approach for defining a process for range camera characterization. Future work for generating 3D versions of other 2D ISO targets has to be performed, in order to enhance the number of quantitative data obtainable with simple test objects and procedures. At the moment, this methodology seems to give usable results for verifying instruments performances in standard laboratory activities.

## Figures and Tables

**Table 1.** Nominal characteristics of the instruments used for the tests, at the chosen working distances, available from the producers data sheets (all values are in mm).

**Range sensor**	**Focal length**	**Working Distance**	**Sample spacing**	**Accuracy**	**Uncertainty**
NextEngine	Not Avail.	430	0.4 × 0.4	0.38	Not avail.
GOM Atos II	17	750	0.3 × 0.3	0.03	Not avail.
Minolta VI-9i	14	750	0.4 × 0.4	0.13	0.02
Minolta VI-9i	25	1200	0.3 × 0.3	0.12	0.03
Minolta VI-910	14	750	0.4 × 0.4	0.30	0.07
Minolta VI-910	25	1200	0.4 × 0.4	0.30	0.08
ShapeGrabber SG 1000	Not Avail.	350	0.2 × 0.3	0.16	0.03
ShapeGrabber SG 100	Not Avail.	200	0.1 × 0.3	0.05	0.02
Minolta Range 7	8	760	0.1 × 0.1	0.06	0.01

**Table 2.** Nominal and actual step size of the “set of steps” test object shown in Figure 3a.

	**Steps size (μm)**									
**Nominal value**	15	30	60	120	240	480	960	1920	3840	7680
**CMM measured value**	16	32	60	120	239	480	960	1920	3838	7672

**Table 3.** Plane fitting parameters obtained from each analyzed range sensors; σ_1_ is the standard deviation evaluated on the whole range map while σ _2_ on the 50 mm × 50 mm central area.

**Range sensor (focal length)**	**Area (mm × mm)**	**σ_1_ (μm)**	**Δz_max_ (μm)**	**Δz_min_ (μm)**	**σ_2_ (μm)**
NextEngine	137 × 125	77	390	−485	35.2
GOM Atos II	360 × 290	18	55	−146	4.6
Minolta VI-9i (14 mm)	247 × 191	45	294	−106	18.5
Minolta VI-9i (25 mm)	208 × 165	61	366	−297	35.9
Minolta VI-910 (14 mm)	251 × 188	66	427	−443	46.4
Minolta VI-910 (25 mm)	220 × 167	133	354	−532	59.9
ShapeGrabber SG1000	527 × 238	164	734	−490	27.3
ShapeGrabber SG100	435 × 117	53	194	−348	28.8
Minolta Range7	320 × 255	28	191	−197	8.4

**Table 4.** Uncertainty related to the different geometries analyzed in the set of solids.

**Range camera (focal length)**	**σ (μm)**			
	**Upper plane**	**Lower plane**	**Cylinder (average)**	**Cone (average)**
NextEngine	33	29	114	53
GOM Atos II	15	18	159	74
Minolta VI-9i (14 mm)	39	33	97	36
Minolta VI-9i (25 mm)	90	76	95	134
Minolta VI-910 (14 mm)	47	49	68	97
Minolta VI-910 (25 mm)	64	56	97	327
ShapeGrabber SG1000	83	65	116	52
ShapeGrabber SG100	26	25	85	50
Minolta Range7	16	21	18	18

**Table 5.** Relative linear accuracy evaluated on the range devices under test.

**Range camera (focal length)**	**Absolute error (μm)**	**Image diagonal (mm)**	**A_LR_ (μm/mm)**
NextEngine	875	185.5	4.72
GOM Atos II	201	462.3	0.31
Minolta VI-9i (14 mm)	400	312.2	1.28
Minolta VI-9i (25 mm)	663	265.5	2.50
Minolta VI-910 (14 mm)	870	313.6	2.77
Minolta VI-910 (25 mm)	886	276.2	3.21
ShapeGrabber SG1000	1.224	578.2	2.12
ShapeGrabber SG100	542	450.5	1.20
Minolta Range7	388	409.2	0.95

**Table 6.** Angular and linear accuracy report. Deviation between angles and linear dimensions (Radius and Height) evaluated by means of the CMM equipment, and corresponding angles and dimensions evaluated from planes fitted onto the primitives or extracted directly from the range maps.

**Range camera (focal length)**	**Δα (degrees)**	**Δβ (degrees)**	**ΔH (μm)**	**ΔR (μm)**
NextEngine	0.11	0.02	−221	−12
GOM Atos II	0.11	0.01	16	44
Minolta VI-9i (14 mm)	−0.04	−0.06	761	54
Minolta VI-9i (25 mm)	−0.39	−0.07	547	−32
Minolta VI-910 (14 mm)	0.24	−0.01	152	11
Minolta VI-910 (25 mm)	0.09	0.03	−462	−49
ShapeGrabber SG1000	−0.06	−0.02	738	165
ShapeGrabber SG100	0.01	0.07	−530	41
Minolta Range7	−0.03	0.02	−156	1

**Table 7.** Step-to-reference distance deviation. RMS distance between the plane located at the distance from the bottom given by the CMM measurement and the corresponding set of data originated by the range camera. Some of the cells in the table have not been filled due to the undistinguishbility between adjacent sets of data according to the definition given in section 6.2. Step 1 is the smaller (16 μm).

**Range camera (focal length)**	**Deviation (μm) at the different steps**										**Mean (μm)**
	**1**	**2**	**3**	**4**	**5**	**6**	**7**	**8**	**9**	**10**	
NextEngine	/	/	/	/	3	6	30	16	49	−35	12
GOM Atos II	/	/	18	1	−13	2	−3	−14	9	4	1
Minolta VI-9i (14 mm)	/	/	24	11	2	6	11	24	−6	−2	9
Minolta VI-9i (25 mm)	/	/	14	−2	4	3	28	3	14	26	11
Minolta VI-910 (14 mm)	/	/	13	14	27	28	35	49	56	63	36
Minolta VI-910 (25 mm)	/	/	37	33	47	67	89	9	2	−28	32
ShapeGrabber SG1000	/	/	0	−17	−6	−7	−13	−17	−27	−51	−17
ShapeGrabber SG100	−31	−23	−10	−12	−8	13	15	11	24	−1	−2
Minolta Range7	7	6	6	3	4	3	3	7	10	10	6

**Table 8.** Values of MTF50 for the spectra shown in Figure 12.

	**MTF50 (cy/px)**

**No blur**	1.063
**Blur 0.5**	0.247
**Blur 1.0**	0.155
**Blur 2.0**	0.087

**Table 9.** Values of MTF50 for the different scanners analyzed in the paper, whose spectra are reported in Figure 13.

**Scanner type**	**MTF50 (cy/mm)**
NextEngine (Wide)	NA
GOM Atos II	1.65
Minolta VI-9i (Middle)	0.64
Minolta VI-9i (Tele)	1.35
Minolta VI-910 (Middle)	0.65
Minolta VI-910 (Tele)	1.63
ShapeGrabber SG1000	0.19
ShapeGrabber SG100	0.78
Minolta Range7	NA
